# Cancer Screening Practices Among Healthcare Workers During the COVID-19 Pandemic

**DOI:** 10.3389/fpubh.2022.801805

**Published:** 2022-03-14

**Authors:** Geetanjali D. Datta, Marie Lauzon, Sarah-Jeanne Salvy, Shehnaz K. Hussain, Sara Ghandehari, Akil Merchant, Noah M. Merin, Karen Reckamp, Jane C. Figueiredo

**Affiliations:** ^1^Department of Medicine, Samuel Oschin Comprehensive Cancer Institute, Cedars-Sinai Medical Center, Los Angeles, CA, United States; ^2^Department of Public Health Sciences, University of California (UC) Davis School of Medicine and Comprehensive Cancer Center, Davis, CA, United States; ^3^Cedars-Sinai Medical Center, Pulmonary Rehabilitation in the Women's Guild Lung Institute, Los Angeles, CA, United States; ^4^Division of Hematology and Cellular Therapy, Samuel Oschin Comprehensive Cancer Institute, Cedars-Sinai Medical Center, Los Angeles, CA, United States

**Keywords:** healthcare workers, cancer screening, lung cancer, breast cancer, colorectal cancer

## Abstract

The COVID-19 pandemic has the potential to impact long-standing efforts to increase adherence to cancer screening guidelines. Healthcare workers (HCWs) experienced significant hardship, but generally have greater access to preventive services, making them a particularly relevant population in which to understand cancer screening behaviors during the pandemic. We report data from 794 HCWs enrolled in the NCI-funded Serological Sciences Network for Coronavirus Associations and Longitudinal Evaluation Study from December 2020 to April 2021. Participants reported lifestyle and screening behaviors during relevant look-back periods which included the pandemic timeframe. Among women between the ages of 40 and 74, 25.7% were overdue for mammographic breast cancer screening. Among participants 50–75 years old, 38.9% were overdue for colorectal cancer screening. The proportion over-due varied according to race/ethnicity. Lifetime low-dose computed tomography lung cancer screening among HCWs age 50–80 years who were smokers was 10.9%. Strategies to address screening disruptions are needed to minimize the impact of later stage of diagnosis.

## Introduction

Health care services across the globe have been profoundly affected by the COVID-19 pandemic. The suspension of cancer screening programs, cancellation or rescheduling of non-elective procedures, a pivot toward virtual clinical visits, and stay-at-home orders may have impacted adherence to cancer screening (1, 2). Data from one population-based surveillance program in the United States (U.S.) noted a reduction in the number of breast examinations and screening mammograms during the pandemic, with the greatest lag in rebound visits among Asian and Hispanic women (3). These findings underlie the importance of understanding screening practices during the pandemic and their possible impact in exacerbating inequalities. Such information can prepare health care systems to react appropriately with “catch-up” strategies to mitigate the potential long-term cascade effects.

## Methods

Healthcare workers (HCW) experienced significant hardship during the pandemic (4), but generally have greater access to preventive services, making them a particularly relevant population in which to understand cancer screening behaviors during this timeframe. We report data from the U.S. National Cancer Institute-funded Serological Sciences Network for Coronavirus Associations and Longitudinal Evaluation Study (CORALE). The SeroNet-CORALE study is a longitudinal prospective cohort including HCWs at a large health care system in the diverse metropolis of Los Angeles that began in November 2020.

### Study Sample

Active employees working at multiple sites comprising the Cedars-Sinai Health System, located in the diverse metropolis of Los Angeles County, California were eligible to participate. The Cedars-Sinai organization includes two hospitals (Cedars-Sinai Medical Center and Marina del Rey Hospital) in addition to multiple clinics in the Cedars-Sinai Medical Delivery Network. The SeroNet-CORALE Study recruited HCWs from vaccine clinics at Cedars-Sinai Medical Center and through study notifications in mass emails between December 2020 and April 2021. Participants were invited to complete questionnaires, donate blood specimens and engage in long-term follow-up. Cancer screening behaviors were measured using modified versions of standardized questionnaires (5, 6). We report data on participants age 40 and older (*N* = 794). The healthcare workers represent employees with diverse job functions and the sample was composed of 37% nurses, 32% physicians, 5% medical technicians, 4% hospital administration, 2% nursing assistant, and 20% from various other occupations.

### Measures

Through the study survey, participants were asked “Have you had any of the following screenings?” Screening tests and accompanying details were listed, including “Mammogram (an x-ray of each breast to look for breast cancer),” “blood stool test (a special kit you use at home where you have a bowel movement and use a stick to smear a small sample on a special card to determine whether the stool contains blood),” “sigmoidoscopy (exam in which a tube is inserted in the rectum to view the colon for signs of cancer or other health problems),” “colonoscopy (exam similar to the sigmoidoscopy but uses a longer tube, and you are usually given medication through a needle in your arm to make you sleep and told to have someone else drive you home after the test),” “CT or CAT scan (during this test, you lie flat on your back on a table. While you hold your breath, the table moves through a donut shaped x-ray machine while the scan is done).” Participants were able to respond “yes,” “no,” or “not sure” to each of the screening tests listed.

The U.S. Preventive Services Task Force (USPSTF) recommends biennial screening with conventional mammography for women aged 50–74 years. Screening between ages 40–49 is considered an individual decision and there is no recommendation over the age of 75 years (7). Women were considered “over-due” for breast cancer screening if they were ≥40 years of age and they had not received a mammogram in the 2 years previous to the completion of the study survey. Duration since last mammogram was ascertained *via* a survey question that asked “How long has it been since you had your last mammogram?” The response categories included “within the past year (anytime <12 months ago),” “within the past 2 years (1 year but <2 years ago),” “within the past 3 years (2 years but <3 years),” “within the past 5 years (3 years but <5 years ago),” “5 or more years ago,” “never,” “not sure.”

For early detection of colorectal cancer, current recommendations by the USPSTF offer several screening modalities depending on preferences (8). We defined over-due as no reported screening *via* stool-based test in the past year, or flexible sigmoidoscopy or colonoscopy within 5 years. Duration since colorectal cancer screening was ascertained *via* three separate questions for each screening modality that asked “How long has it been since you have your last blood stool test/sigmoidoscopy/colonoscopy?” Response categories included “within the past year (anytime <12 months ago),” “within the past 2 years (1 year but <2 years ago),” “within the past 3 years (2 years but <3 years),” “within the past 5 years (3 years but <5 years ago),” “5 or more years ago,” “never,” “not sure.” The screening nature of the exams were ascertained *via* three questions which asked “Why did you have a blood stool test/sigmoidoscopy/colonoscopy?” Participants were instructed to “select all that apply” for the following response categories “Family history of colorectal cancer,” “part of regular check-up/routine screening,” “age,” “race,” follow-up on a problem,” “follow-up of colorectal cancer treatment,” “other (please specify).” Tests were only considered screening tests if the participant selected “part of regular check-up/routine screening.”

At the time of the survey, the USPSTF recommended annual lung cancer screening to those age 50–80 years who had a 20 pack-year smoking history *via* low-dose computed tomography (CT) (9). Guideline recommended annual screening was not assessed in this sample due to the low number of participants who reported smoking 20 or more pack-years (*n* = 6). Instead, the proportion ever screened for lung cancer was calculated for all smokers (former and current combined regardless of pack-years) age 50–80 years. Duration since lung cancer screening was ascertained *via* a question that asked “How long has it been since you had your last CT or CAT scan?” The response categories included “within the past year (anytime <12 months ago),” “within the past 2 years (1 year but <2 years ago),” “within the past 3 years (2 years but <3 years),” “within the past 5 years (3 years but <5 years ago),” “5 or more years ago,” “never,” “not sure.” The screening nature of the exam was ascertained *via* a question that asked “Why did you have a CT or CAT scan?” Participants were instructed to “select all that apply” for the following response categories “part of prior hospitalization,” “part or regular check-up/routine screening,” “follow-up on lung problem,” “follow-up of lung cancer treatment,” “not sure,” “other (please specify).” The test was only considered a screening test if the participant selected “part of regular check-up/routine screening.”

Breast and colorectal cancer screening were examined descriptively by selected patient characteristics including age group and race/ethnicity. Logistic regression models with sociodemographic predictors were implemented for breast and colorectal cancer separately. Because of insufficient sample size, some categories within variables were combined in the models. All analyses were performed using SAS software (SAS, version 9.4, Carey, N.C.). Participants provided informed consent and the study was approved by the Cedars-Sinai IRB.

## Results

Baseline characteristics for the study population are shown in Table 1. Median age at time of enrollment was 51.3 years [interquartile range (IQR): 45.2–61.4 years]. The majority of the study sample were women (57.6%), 18.2% percent of participants had a BMI of 30 or greater, and 19.5% were former or current smokers.

**Table 1:** Descriptive characteristics of the study population.

	**Female** **(*N* = 457)**	**Male** **(*N* = 337)**	**Total** **(*N* = 794)**
**Age**, median [IQR]	51.4 [44.5, 59.9]	51.1 [45.7, 63.5]	51.3 [45.2, 61.4]
**Race/Ethnicity**			
Missing	31	39	70
Hispanic	46 (10.8%)	34 (11.4%)	80 (11.0%)
Non-Hispanic Asian	108 (25.4%)	71 (23.8%)	179 (24.7%)
Non-Hispanic black	24 (5.6%)	4 (1.3%)	28 (3.9%)
Non-Hispanic white	207 (48.6%)	165 (55.4%)	372 (51.4%)
Other	41 (9.6%)	24 (8.1%)	65 (9.0%)
**Education**			
Missing	57	63	120
< High school	3 (0.8%)	5 (1.8%)	8 (1.2%)
High school graduate	5 (1.3%)	6 (2.2%)	11 (1.6%)
Some college	45 (11.3%)	31 (11.3%)	76 (11.3%)
College graduate	89 (22.3%)	33 (12.0%)	122 (18.1%)
Some school beyond college/Graduate degree	258 (64.5%)	199 (72.6%)	457 (67.8%)
**Income**			
Missing	61	66	127
< $75,000	59 (14.9%)	29 (10.7%)	88 (13.2%)
$75,000 or more	286 (72.2%)	207 (76.4%)	493 (73.9%)
Not sure	7 (1.8%)	3 (1.1%)	10 (1.5%)
Prefer not to answer	44 (11.1%)	32 (11.8%)	76 (11.4%)
**BMI**			
Missing	48	49	97
<18.5	7 (1.7%)	0 (0.0%)	7 (1.0%)
18.5– <25	207 (50.6%)	114 (39.6%)	321 (46.1%)
25– <30	116 (28.4%)	126 (43.8%)	242 (34.7%)
30+	79 (19.3%)	48 (16.7%)	127 (18.2%)
**Smoking status**			
Missing	9	14	23
Current	5 (1.1%)	15 (4.6%)	20 (2.6%)
Former	79 (17.6%)	51 (15.8%)	130 (16.9%)
Never	364 (81.3%)	257 (79.6%)	621 (80.5%)

^*^Percentages exclude missing data.

Among women between the ages of 40 and 74 (*N* = 381), 25.7% were over-due for breast cancer screening (Table 2). A smaller proportion of non-Hispanic Black (17.4%) and non-Hispanic White (18.3%) women were over-due for breast cancer screening compared to non-Hispanic Asian (36.3%) or Hispanic (33.3%) women. In bivariate and multivariate models, race/ethnicity was statistically significantly associated with breast cancer screening with non-Hispanic White women being less likely to be overdue for screening than other women [OR_adj_ = 0.54: 95% confidence interval (CIs) 0.31–0.94] (Table 3).

**Table 2:** Cancer screening rates and adherence to guidelines for breast, lung and colorectal cancers^*^.

**Breast cancer screening**		**Colorectal cancer screening**		**Lung cancer screening**	
**Characteristic**	**Overdue**	**Characteristic**	**Overdue**	**Characteristic**	**Low-dose CT (lifetime)**
Age		Age			
40–74 y (*n* = 381)	98 (25.7%)	45–49 y (*n* = 143)	122 (85.3%)	All smokers (*n* = 150)	13 (8.7%)
>74 y (*n* = 11)	3 (27.3)	50–75 y (*n* = 350)	136 (38.9%)	50–80 y smokers (*n* = 92)	10 (10.9%)
		≥76 y (*n* = 15)	8 (53.3%)		
Ethnicity/Race (40–74 y)		Ethnicity/Race (50–75 y)		Ethnicity/Race (50–80 y smokers)	
Hispanic (*n* = 39)	13 (33.3%)	Hispanic (*n* = 24)	6 (25.0%)	Hispanic (*n* = 3)	0 (0.0%)
Non-Hispanic Asian (*n* = 102)	37 (36.3%)	Non-Hispanic Asian (*n* = 72)	27 (37.5%)	Non-Hispanic Asian (*n* = 11)	4 (36.4%)
Non-Hispanic black (*n* = 23)	4 (17.4%)	Non-Hispanic black (*n* = 19)	12 (63.2%)	Non-Hispanic black (*n* = 4)	1 (25.0%)
Non-Hispanic white (*n* = 180)	33 (18.3%)	Non-Hispanic white (*n* = 209)	77 (36.8%)	Non-Hispanic white (*n* = 54)	5 (9.3%)
Other (*n* = 36)	11 (30.6%)	Other (*n* = 22)	12 (54.5%)	Other (*n* = 4)	0 (0.0%)

^*^Percentages exclude missing data.

**Table 3:** Univariate and fully-adjusted logistic regression models of over-due breast cancer screening.

	**Univariate**		**Multivariable**	
	**OR**	**95% CI lower, upper**	**OR**	**95% CI lower, upper**
**Age**	0.93	0.90, 0.95	0.92	0.89, 0.96
**Race**				
White	0.49	0.31, 0.78	0.54	0.31, 0.94
Non-White	1		1	
**Education**				
< High school, High school graduate, or Some college	1		1	
College graduate	1.07	0.50, 2.28	1.07	0.42, 2.74
Some school beyond college or Graduate degree	0.78	0.40, 1.51	0.82	0.35, 1.92
**Income**				
$75,000 or more	1.17	0.60, 2.25	2.12	0.94, 4.80
< $75,000	1		1	
**BMI**				
<25	1		1	
25– <30	0.88	0.50, 1.56	0.87	0.45, 1.69
30+	1.79	1.00, 3.20	1.92	0.98, 3.76
**Smoking**				
Ever smoker	0.79	0.43, 1.45	0.81	0.38, 1.73
Never smoker	1		1	

For colorectal cancer screening, among those in the 50-75 screening age (*N* = 350), 38.9% were over-due for screening. The proportion over-due among Hispanic (25.0%), non-Hispanic Asian (37.5%), and non-Hispanic White (36.8%). HCWs was lower than those among non-Hispanic Black (63.2%) HCWs. In bivariate and multivariate models, none of the predictors were statistically significantly associated with colorectal cancer screening (Table 4).

**Table 4:** Univariate and fully-adjusted logistic regression models of over-due colorectal cancer screening.

	**Univariate**		**Multivariable**	
	**OR**	**95% CI lower, upper**	**OR**	**95% CI lower, upper**
**Gender**				
Female	1		1	
Male	1.08	0.76, 1.53	1.03	0.66, 1.60
**Age**	0.91	0.89, 0.93	0.91	0.88, 0.93
**Race**				
White	0.70	0.49, 1.00	0.95	0.61, 1.49
Non-White	1		1	
**Education**				
< High school, High school graduate, or Some college	1		1	
College graduate	0.77	0.40, 1.50	0.74	0.32, 1.74
Some school beyond college, or Graduate degree	0.64	0.37, 1.10	0.61	0.29, 1.28
**Income**				
$75,000 or more	0.77	0.44, 1.35	0.95	0.49, 1.83
< $75,000	1		1	
**BMI**				
<25	1		1	
25– <30	1.16	0.77, 1.72	1.32	0.81, 2.17
30+	1.49	0.92, 2.41	1.47	0.82, 2.66
**Smoking**				
Ever smoker	0.92	0.59, 1.43	1.06	0.61, 1.86
Never smoker	1		1	

Among men and women age 50–80 years who were either current or former smokers (*N* = 92), lifetime CT lung cancer screening was 10.9%. Lifetime screening was lowest in Hispanics compared to other ethnic/racial groups.

## Discussion

In this study of healthcare workers with a screening look-back period that included healthcare disruptions due to COVID-19, we observed that 25.7% of women between the ages of 40 and 74 years of age were over-due for breast cancer screening and 38.9% of people between the ages of 50 and 75 years of age were overdue for colorectal cancer screening. Additionally, among current or former smokers smokers between the ages of 50 and 80 years old 10.9% had ever received a screening CT exam. To our knowledge this is the first report of cancer screening practices among HCWs during the COVID-19 pandemic in the U.S. An important strength of our study is the ability to examine screening behaviors among healthcare workers across ethnic/racial categories.

These data highlight similar over-due rates for breast screening during the pandemic as reported in pre-pandemic population samples (~27%) (10). The proportion over-due for CRC screening was slightly higher in the current study (38.9 vs. 31% in national samples) (11). This difference could have been due to differences in definitions for adherence, study population, or reflect screening behavior changes due to COVID-19 disruptions. Importantly, even in this population with access to preventive health care, variations by ethnicity and race were observed.

### Limitations

Limitations of our analysis include missing data and the cross-sectional nature of the study. Our sample size for lung cancer screening is also limited by the small number of HCWs who smoked.

## Conclusion

These results have important public health implications. Effective communication about how the pandemic has affected cancer screening, in particular minority populations is needed. Use of home-based stool tests for colorectal cancer could also help “catch-up” strategies (12). More research is needed to explore best strategies for suspending, resuming and sustaining cancer screening programs, and preparedness for future disruptions, adapted to diverse populations and health care systems in order to prevent later stage of diagnosis and its sequalae.

## Data Availability Statement

The raw data supporting the conclusions of this article will be made available by the authors, without undue reservation.

## Ethics Statement

The studies involving human participants were reviewed and approved by Cedars Sinai Institutional Review Board. The patients/participants provided their written informed consent to participate in this study.

## Author Contributions

GD: conceptualization, methodology, supervision, writing—original draft, and writing—review and editing. ML: data curation, formal analysis, methodology, writing—original draft, and writing—review and editing. S-JS: methodology, writing—original draft, and writing—review and editing. SH: funding acquisition, methodology, writing—original draft, and writing—review and editing. SG: writing—review and editing. AM and NM: funding acquisition, resources, and writing—review and editing. KR: funding acquisition, resources, methodology, writing—original draft, and writing—review and editing. JF: conceptualization, funding acquisition, investigation, resources, supervision, writing—original draft, and writing—review and editing. All authors contributed to the article and approved the submitted version.

## Funding

This work was supported by the National Cancer Institute (U54CA260591). The funder had no role in the execution or reporting of this research study.

## Conflict of Interest

The authors declare that the research was conducted in the absence of any commercial or financial relationships that could be construed as a potential conflict of interest.

## Publisher's Note

All claims expressed in this article are solely those of the authors and do not necessarily represent those of their affiliated organizations, or those of the publisher, the editors and the reviewers. Any product that may be evaluated in this article, or claim that may be made by its manufacturer, is not guaranteed or endorsed by the publisher.
